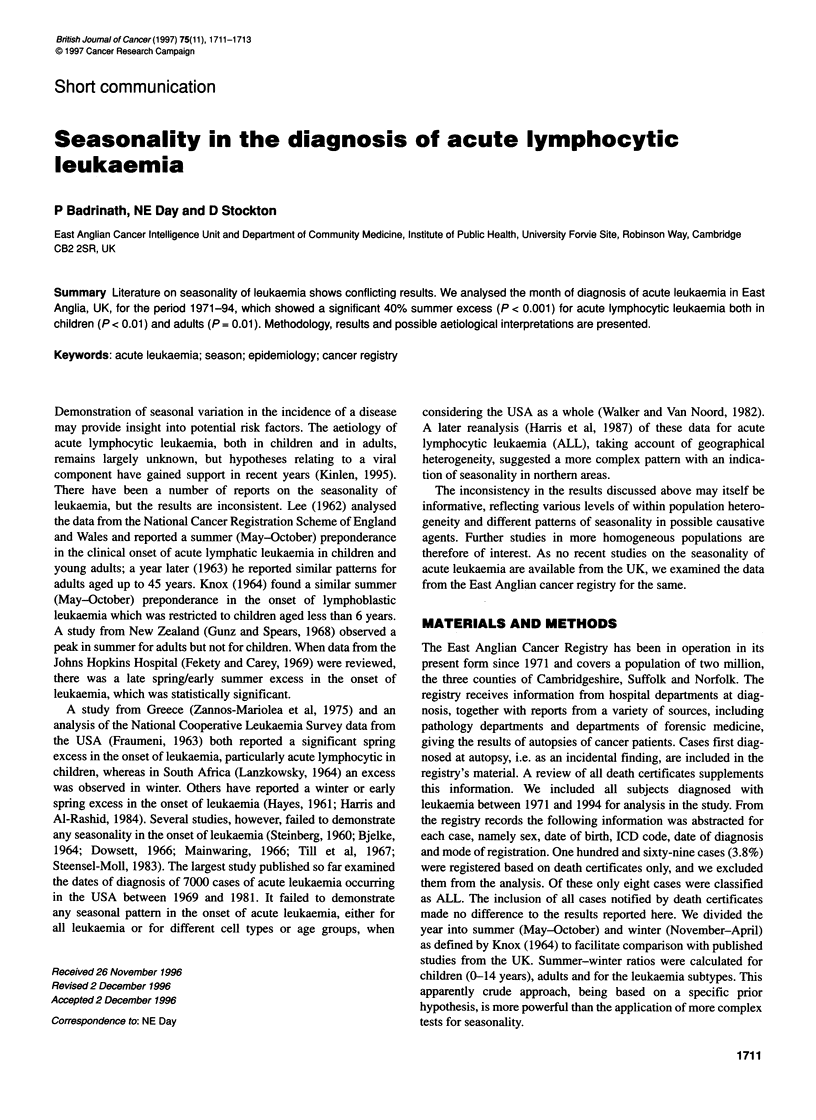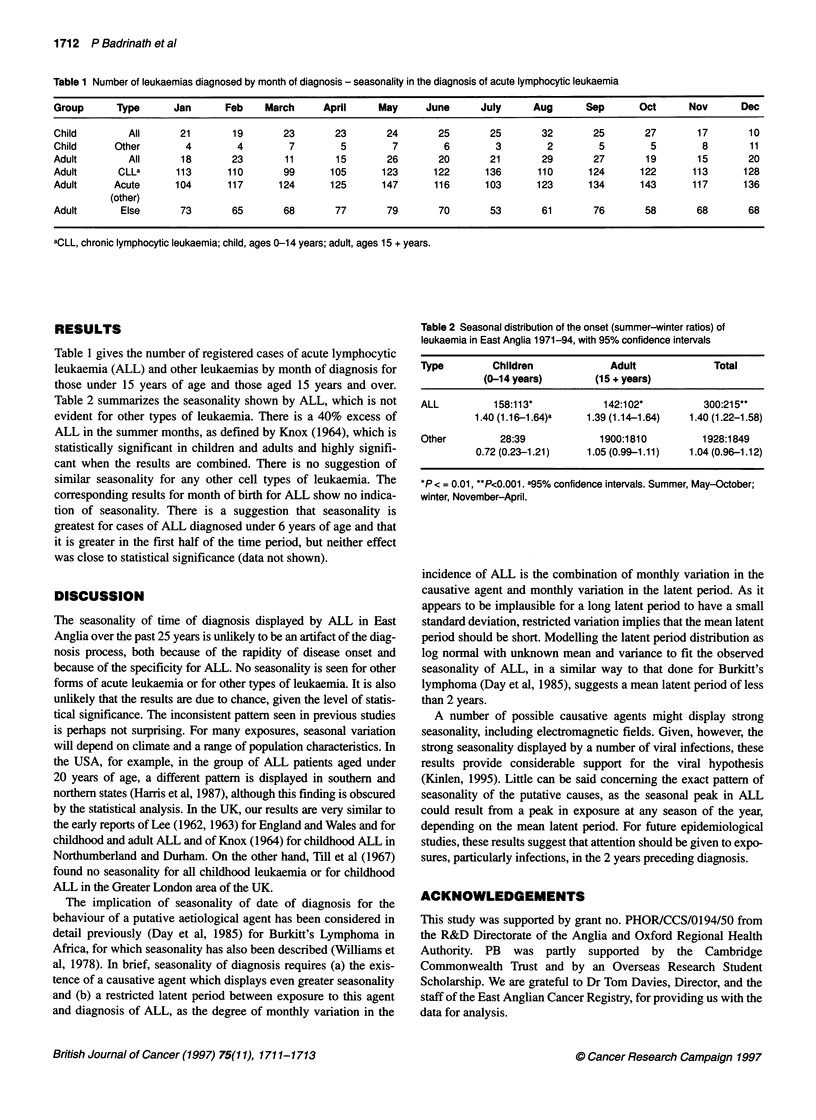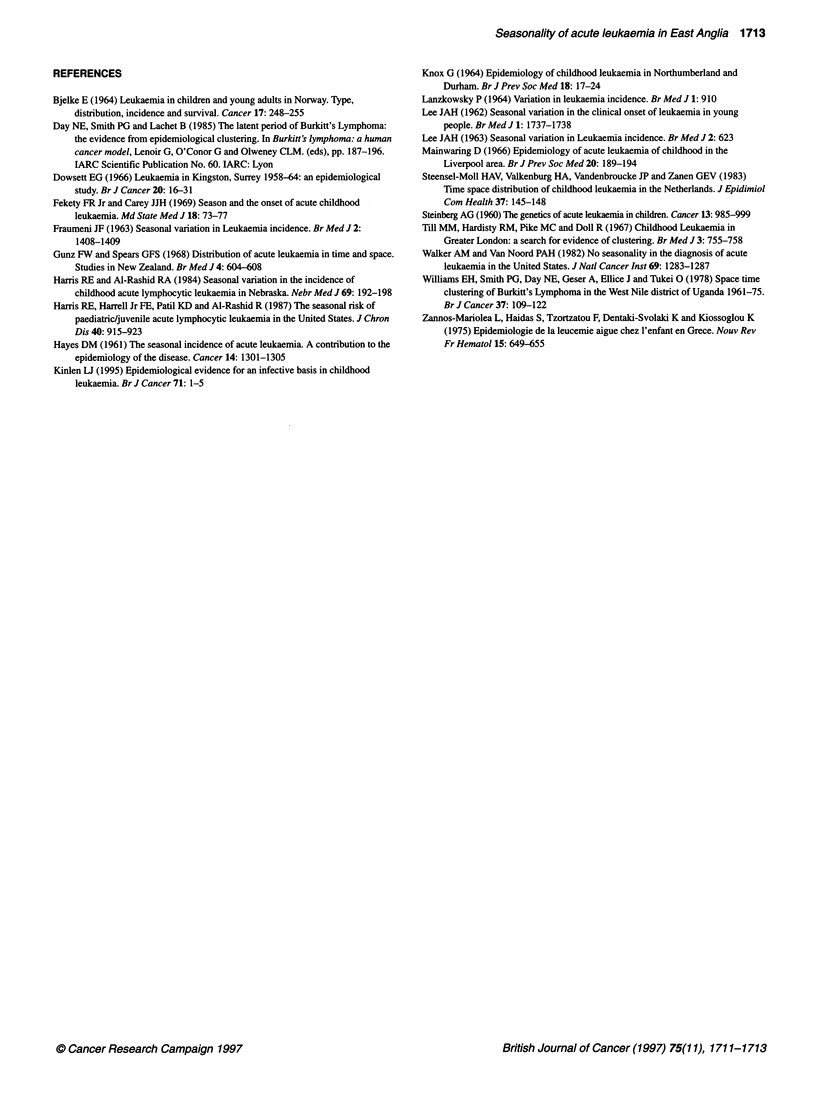# Seasonality in the diagnosis of acute lymphocytic leukaemia.

**DOI:** 10.1038/bjc.1997.292

**Published:** 1997

**Authors:** P. Badrinath, N. E. Day, D. Stockton

**Affiliations:** Department of Community Medicine, Institute of Public Health, University Forvie Site, Cambridge, UK.

## Abstract

Literature on seasonality of leukaemia shows conflicting results. We analysed the month of diagnosis of acute leukaemia in East Anglia, UK, for the period 1971-94, which showed a significant 40% summer excess (P < 0.001) for acute lymphocytic leukaemia both in children (P < 0.01) and adults (P = 0.01). Methodology, results and possible aetiological interpretations are presented.


*British Journal of Cancer* (1997) **75**(11), 1711–1713
© 1997 Cancer Research Campaign

Short communication

### Seasonality in the diagnosis of acute lymphocytic leukaemia

**P Badrinath, NE Day and D Stockton**

East Anglian Cancer Intelligence Unit and Department of Community Medicine, Institute of Public Health, University Forvie Site, Robinson Way, Cambridge CB2 2SR, UK

**Summary** Literature on seasonality of leukaemia shows conflicting results. We analysed the month of diagnosis of acute leukaemia in East Anglia, UK, for the period 1971–94, which showed a significant 40% summer excess ($P < 0.001$) for acute lymphocytic leukaemia both in children ($P < 0.01$) and adults ($P = 0.01$). Methodology, results and possible aetiological interpretations are presented.

**Keywords**: acute leukaemia; season; epidemiology; cancer registry

Demonstration of seasonal variation in the incidence of a disease may provide insight into potential risk factors. The aetiology of acute lymphocytic leukaemia, both in children and in adults, remains largely unknown, but hypotheses relating to a viral component have gained support in recent years (Kinlen, 1995). There have been a number of reports on the seasonality of leukaemia, but the results are inconsistent. Lee (1962) analysed the data from the National Cancer Registration Scheme of England and Wales and reported a summer (May–October) preponderance in the clinical onset of acute lymphatic leukaemia in children and young adults; a year later (1963) he reported similar patterns for adults aged up to 45 years. Knox (1964) found a similar summer (May–October) preponderance in the onset of lymphoblastic leukaemia which was restricted to children aged less than 6 years. A study from New Zealand (Gunz and Spears, 1968) observed a peak in summer for adults but not for children. When data from the Johns Hopkins Hospital (Fekety and Carey, 1969) were reviewed, there was a late spring/early summer excess in the onset of leukaemia, which was statistically significant.

A study from Greece (Zannos-Mariolea et al, 1975) and an analysis of the National Cooperative Leukaemia Survey data from the USA (Fraumeni, 1963) both reported a significant spring excess in the onset of leukaemia, particularly acute lymphocytic in children, whereas in South Africa (Lanzkowsky, 1964) an excess was observed in winter. Others have reported a winter or early spring excess in the onset of leukaemia (Hayes, 1961; Harris and Al-Rashid, 1984). Several studies, however, failed to demonstrate any seasonality in the onset of leukaemia (Steinberg, 1960; Bjelke, 1964; Dowsett, 1966; Mainwaring, 1966; Till et al, 1967; Steensel-Moll, 1983). The largest study published so far examined the dates of diagnosis of 7000 cases of acute leukaemia occurring in the USA between 1969 and 1981. It failed to demonstrate any seasonal pattern in the onset of acute leukaemia, either for all leukaemia or for different cell types or age groups, when considering the USA as a whole (Walker and Van Noord, 1982). A later reanalysis (Harris et al, 1987) of these data for acute lymphocytic leukaemia (ALL), taking account of geographical heterogeneity, suggested a more complex pattern with an indication of seasonality in northern areas.

The inconsistency in the results discussed above may itself be informative, reflecting various levels of within population heterogeneity and different patterns of seasonality in possible causative agents. Further studies in more homogeneous populations are therefore of interest. As no recent studies on the seasonality of acute leukaemia are available from the UK, we examined the data from the East Anglian cancer registry for the same.

### MATERIALS AND METHODS

The East Anglian Cancer Registry has been in operation in its present form since 1971 and covers a population of two million, the three counties of Cambridgeshire, Suffolk and Norfolk. The registry receives information from hospital departments at diagnosis, together with reports from a variety of sources, including pathology departments and departments of forensic medicine, giving the results of autopsies of cancer patients. Cases first diagnosed at autopsy, i.e. as an incidental finding, are included in the registry's material. A review of all death certificates supplements this information. We included all subjects diagnosed with leukaemia between 1971 and 1994 for analysis in the study. From the registry records the following information was abstracted for each case, namely sex, date of birth, ICD code, date of diagnosis and mode of registration. One hundred and sixty-nine cases (3.8%) were registered based on death certificates only, and we excluded them from the analysis. Of these only eight cases were classified as ALL. The inclusion of all cases notified by death certificates made no difference to the results reported here. We divided the year into summer (May–October) and winter (November–April) as defined by Knox (1964) to facilitate comparison with published studies from the UK. Summer–winter ratios were calculated for children (0–14 years), adults and for the leukaemia subtypes. This apparently crude approach, being based on a specific prior hypothesis, is more powerful than the application of more complex tests for seasonality.

*Received 26 November 1996*
*Revised 2 December 1996*
*Accepted 2 December 1996*

*Correspondence to*: NE Day

Table 1 Number of leukaemias diagnosed by month of diagnosis – seasonality in the diagnosis of acute lymphocytic leukaemia

| Group | Type | Jan | Feb | March | April | May | June | July | Aug | Sep | Oct | Nov | Dec |
|---|---|---|---|---|---|---|---|---|---|---|---|---|---|
| Child | All | 21 | 19 | 23 | 23 | 24 | 25 | 25 | 32 | 25 | 27 | 17 | 10 |
| Child | Other | 4 | 4 | 7 | 5 | 7 | 6 | 3 | 2 | 5 | 5 | 8 | 11 |
| Adult | All | 18 | 23 | 11 | 15 | 26 | 20 | 21 | 29 | 27 | 19 | 15 | 20 |
| Adult | CLL[a] | 113 | 110 | 99 | 105 | 123 | 122 | 136 | 110 | 124 | 122 | 113 | 128 |
| Adult | Acute (other) | 104 | 117 | 124 | 125 | 147 | 116 | 103 | 123 | 134 | 143 | 117 | 136 |
| Adult | Else | 73 | 65 | 68 | 77 | 79 | 70 | 53 | 61 | 76 | 58 | 68 | 68 |

[a]CLL, chronic lymphocytic leukaemia; child, ages 0–14 years; adult, ages 15 + years.

### RESULTS

Table 1 gives the number of registered cases of acute lymphocytic leukaemia (ALL) and other leukaemias by month of diagnosis for those under 15 years of age and those aged 15 years and over. Table 2 summarizes the seasonality shown by ALL, which is not evident for other types of leukaemia. There is a 40% excess of ALL in the summer months, as defined by Knox (1964), which is statistically significant in children and adults and highly significant when the results are combined. There is no suggestion of similar seasonality for any other cell types of leukaemia. The corresponding results for month of birth for ALL show no indication of seasonality. There is a suggestion that seasonality is greatest for cases of ALL diagnosed under 6 years of age and that it is greater in the first half of the time period, but neither effect was close to statistical significance (data not shown).

Table 2 Seasonal distribution of the onset (summer–winter ratios) of leukaemia in East Anglia 1971–94, with 95% confidence intervals

| Type | Children (0–14 years) | Adult (15 + years) | Total |
|---|---|---|---|
| ALL | 158:113* | 142:102* | 300:215** |
| | 1.40 (1.16–1.64)[a] | 1.39 (1.14–1.64) | 1.40 (1.22–1.58) |
| Other | 28:39 | 1900:1810 | 1928:1849 |
| | 0.72 (0.23–1.21) | 1.05 (0.99–1.11) | 1.04 (0.96–1.12) |

*$P <= 0.01$, **$P<0.001$. [a]95% confidence intervals. Summer, May–October; winter, November–April.

### DISCUSSION

The seasonality of time of diagnosis displayed by ALL in East Anglia over the past 25 years is unlikely to be an artifact of the diagnosis process, both because of the rapidity of disease onset and because of the specificity for ALL. No seasonality is seen for other forms of acute leukaemia or for other types of leukaemia. It is also unlikely that the results are due to chance, given the level of statistical significance. The inconsistent pattern seen in previous studies is perhaps not surprising. For many exposures, seasonal variation will depend on climate and a range of population characteristics. In the USA, for example, in the group of ALL patients aged under 20 years of age, a different pattern is displayed in southern and northern states (Harris et al, 1987), although this finding is obscured by the statistical analysis. In the UK, our results are very similar to the early reports of Lee (1962, 1963) for England and Wales and for childhood and adult ALL and of Knox (1964) for childhood ALL in Northumberland and Durham. On the other hand, Till et al (1967) found no seasonality for all childhood leukaemia or for childhood ALL in the Greater London area of the UK.

The implication of seasonality of date of diagnosis for the behaviour of a putative aetiological agent has been considered in detail previously (Day et al, 1985) for Burkitt's Lymphoma in Africa, for which seasonality has also been described (Williams et al, 1978). In brief, seasonality of diagnosis requires (a) the existence of a causative agent which displays even greater seasonality and (b) a restricted latent period between exposure to this agent and diagnosis of ALL, as the degree of monthly variation in the incidence of ALL is the combination of monthly variation in the causative agent and monthly variation in the latent period. As it appears to be implausible for a long latent period to have a small standard deviation, restricted variation implies that the mean latent period should be short. Modelling the latent period distribution as log normal with unknown mean and variance to fit the observed seasonality of ALL, in a similar way to that done for Burkitt's lymphoma (Day et al, 1985), suggests a mean latent period of less than 2 years.

A number of possible causative agents might display strong seasonality, including electromagnetic fields. Given, however, the strong seasonality displayed by a number of viral infections, these results provide considerable support for the viral hypothesis (Kinlen, 1995). Little can be said concerning the exact pattern of seasonality of the putative causes, as the seasonal peak in ALL could result from a peak in exposure at any season of the year, depending on the mean latent period. For future epidemiological studies, these results suggest that attention should be given to exposures, particularly infections, in the 2 years preceding diagnosis.

### ACKNOWLEDGEMENTS

This study was supported by grant no. PHOR/CCS/0194/50 from the R&D Directorate of the Anglia and Oxford Regional Health Authority. PB was partly supported by the Cambridge Commonwealth Trust and by an Overseas Research Student Scholarship. We are grateful to Dr Tom Davies, Director, and the staff of the East Anglian Cancer Registry, for providing us with the data for analysis.

© Cancer Research Campaign 1997

### REFERENCES

Bjelke E (1964) Leukaemia in children and young adults in Norway. Type, distribution, incidence and survival. *Cancer* **17**: 248–255

Day NE, Smith PG and Lachet B (1985) The latent period of Burkitt's Lymphoma: the evidence from epidemiological clustering. In *Burkitt's lymphoma: a human cancer model*, Lenoir G, O'Conor G and Olweney CLM. (eds), pp. 187–196. IARC Scientific Publication No. 60. IARC: Lyon

Dowsett EG (1966) Leukaemia in Kingston, Surrey 1958–64: an epidemiological study. *Br J Cancer* **20**: 16–31

Fekety FR Jr and Carey JJH (1969) Season and the onset of acute childhood leukaemia. *Md State Med J* **18**: 73–77

Fraumeni JF (1963) Seasonal variation in Leukaemia incidence. *Br Med J* **2**: 1408–1409

Gunz FW and Spears GFS (1968) Distribution of acute leukaemia in time and space. Studies in New Zealand. *Br Med J* **4**: 604–608

Harris RE and Al-Rashid RA (1984) Seasonal variation in the incidence of childhood acute lymphocytic leukaemia in Nebraska. *Nebr Med J* **69**: 192–198

Harris RE, Harrell Jr FE, Patil KD and Al-Rashid R (1987) The seasonal risk of paediatric/juvenile acute lymphocytic leukaemia in the United States. *J Chron Dis* **40**: 915–923

Hayes DM (1961) The seasonal incidence of acute leukaemia. A contribution to the epidemiology of the disease. *Cancer* **14**: 1301–1305

Kinlen LJ (1995) Epidemiological evidence for an infective basis in childhood leukaemia. *Br J Cancer* **71**: 1–5

Knox G (1964) Epidemiology of childhood leukaemia in Northumberland and Durham. *Br J Prev Soc Med* **18**: 17–24

Lanzkowsky P (1964) Variation in leukaemia incidence. *Br Med J* **1**: 910

Lee JAH (1962) Seasonal variation in the clinical onset of leukaemia in young people. *Br Med J* **1**: 1737–1738

Lee JAH (1963) Seasonal variation in Leukaemia incidence. *Br Med J* **2**: 623

Mainwaring D (1966) Epidemiology of acute leukaemia of childhood in the Liverpool area. *Br J Prev Soc Med* **20**: 189–194

Steensel-Moll HAV, Valkenburg HA, Vandenbroucke JP and Zanen GEV (1983) Time space distribution of childhood leukaemia in the Netherlands. *J Epidimiol Com Health* **37**: 145–148

Steinberg AG (1960) The genetics of acute leukaemia in children. *Cancer* **13**: 985–999

Till MM, Hardisty RM, Pike MC and Doll R (1967) Childhood Leukaemia in Greater London: a search for evidence of clustering. *Br Med J* **3**: 755–758

Walker AM and Van Noord PAH (1982) No seasonality in the diagnosis of acute leukaemia in the United States. *J Natl Cancer Inst* **69**: 1283–1287

Williams EH, Smith PG, Day NE, Geser A, Ellice J and Tukei O (1978) Space time clustering of Burkitt's Lymphoma in the West Nile district of Uganda 1961–75. *Br J Cancer* **37**: 109–122

Zannos-Mariolea L, Haidas S, Tzortzatou F, Dentaki-Svolaki K and Kiossoglou K (1975) Epidemiologie de la leucemie aigue chez l'enfant en Grece. *Nouv Rev Fr Hematol* **15**: 649–655

© Cancer Research Campaign 1997